# Lymphatic metastasis of papillary thyroid carcinoma: mechanism and clinicopathological physiology

**DOI:** 10.3389/fendo.2025.1725077

**Published:** 2026-01-28

**Authors:** Pu Liu, Xianqiang Yu

**Affiliations:** 1 Department of General Surgery, Qingdao Municipal Hospital (Qingdao Hospital of Rehabilitation University), Qingdao, China; 2 Department of Thyroid Disease Diagnosis and Treatment, Qingdao Municipal Hospital (Qingdao Hospital of Rehabilitation University), Qingdao, China

**Keywords:** papillary thyroid carcinoma, lymphatic metastasis, lymph node dissection, BRAF mutation, radioactive iodine

## Abstract

Lymphatic metastasis is a hallmark feature of papillary thyroid carcinoma (PTC), occurring in 30–80% of patients and significantly influencing clinical management. This review comprehensively examines the biological, anatomical, and clinical characteristics of lymphatic spread in PTC, focusing on its diagnostic and therapeutic implications. We detail the molecular mechanisms driving lymphangiogenesis, including the VEGF-C/VEGFR-3 axis and immune-evasion pathways, and highlight the distinct patterns of regional lymph node involvement—from central compartment (Level VI) to lateral (Levels II–V) and rare skip metastases. High-risk clinicopathological features, such as tumor size >2 cm, extrathyroidal extension, and aggressive histological variants, are discussed alongside molecular markers (BRAF V600E, TERT, RET/PTC) that predict metastatic potential. Management strategies are reviewed, balancing the benefits of prophylactic central neck dissection against its risks and emphasizing risk-adapted radioactive iodine therapy. Despite the frequency of lymphatic metastasis, its prognostic impact varies: microscopic nodal disease has minimal effect on survival, while macroscopic or extranodal extension increases recurrence and mortality risks. This synthesis of current evidence aims to guide clinicians in optimizing detection, treatment, and surveillance for PTC patients with lymphatic metastasis.

## 1 Introduction

**Figure 1:**
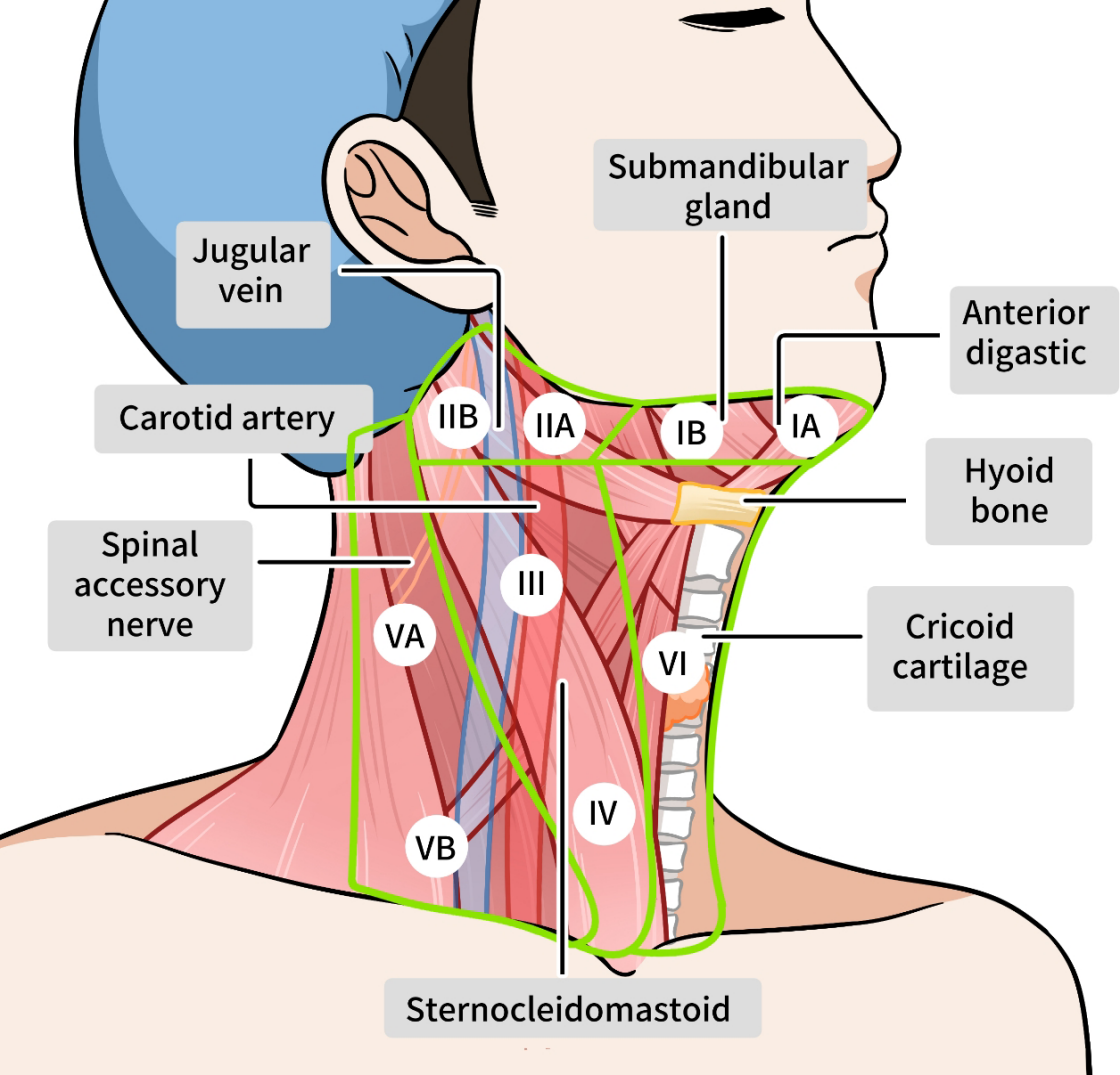
Lymph distribution around the thyroid gland.

Papillary thyroid carcinoma (PTC) demonstrates a unique biological behavior among solid malignancies, characterized by an exceptionally high incidence of lymphatic metastasis that ranges from 30% to 80% in various clinical series, depending on detection methods and patient selection criteria (1–10). This propensity for regional lymph node involvement presents a clinical paradox, as it frequently occurs even in small, intrathyroidal tumors and often does not significantly impact overall survival, yet substantially influences treatment strategies and recurrence risk (11–14). The lymphatic spread typically follows an orderly progression from the central compartment (Level VI) to lateral neck levels (II-V), though approximately 5-20% of cases exhibit “skip metastases” that bypass the central compartment entirely, a phenomenon with significant implications for surgical planning and prognostic assessment (15, 16) (**Figure 1**).

The biological mechanisms underlying lymphatic metastasis in PTC involve a complex interplay of lymphangiogenic factors, particularly the VEGF-C/VEGFR-3 signaling axis, which promotes tumor-associated lymphangiogenesis and facilitates tumor cell entry into lymphatic channels (17, 18). Molecular studies have identified BRAF V600E mutations (present in 40-60% of cases) as strong predictors of lymphatic spread, along with RET/PTC rearrangements (10-20% of cases) that are associated with more extensive nodal involvement (19–22). Recent research has elucidated additional mechanisms contributing to skip metastases, including alternative lymphatic drainage pathways from upper pole tumors and tumor-specific biological properties that may enable direct dissemination to lateral compartments through lymphovenous communications or aberrant lymphatic vessel connections (23–25).

From a clinical perspective, the management of lymphatic metastasis in PTC requires sophisticated diagnostic approaches, including high-resolution ultrasonography (with characteristic findings of round shape, hyperechogenicity, and loss of fatty hilum), thyroglobulin washout analysis of fine-needle aspirates, and selective use of advanced imaging modalities for comprehensive staging (26–28). The presence of skip metastases necessitates particular attention, as these lesions may be missed by routine central compartment evaluation and require modified surgical strategies (29, 30). Current therapeutic paradigms emphasize risk-adapted approaches that consider tumor size, molecular profile, and extent of nodal involvement, while emerging techniques such as lymphatic mapping with indocyanine green fluorescence and targeted therapies against specific metastatic pathways offer promising avenues for personalized management. This review provides a comprehensive analysis of the characteristic features of lymphatic metastasis in PTC, with particular focus on the clinical significance and management implications of skip metastases.

## 2 Lymphatic biology and metastatic mechanisms

### 2.1 Lymphangiogenesis in PTC

**Figure 2:**
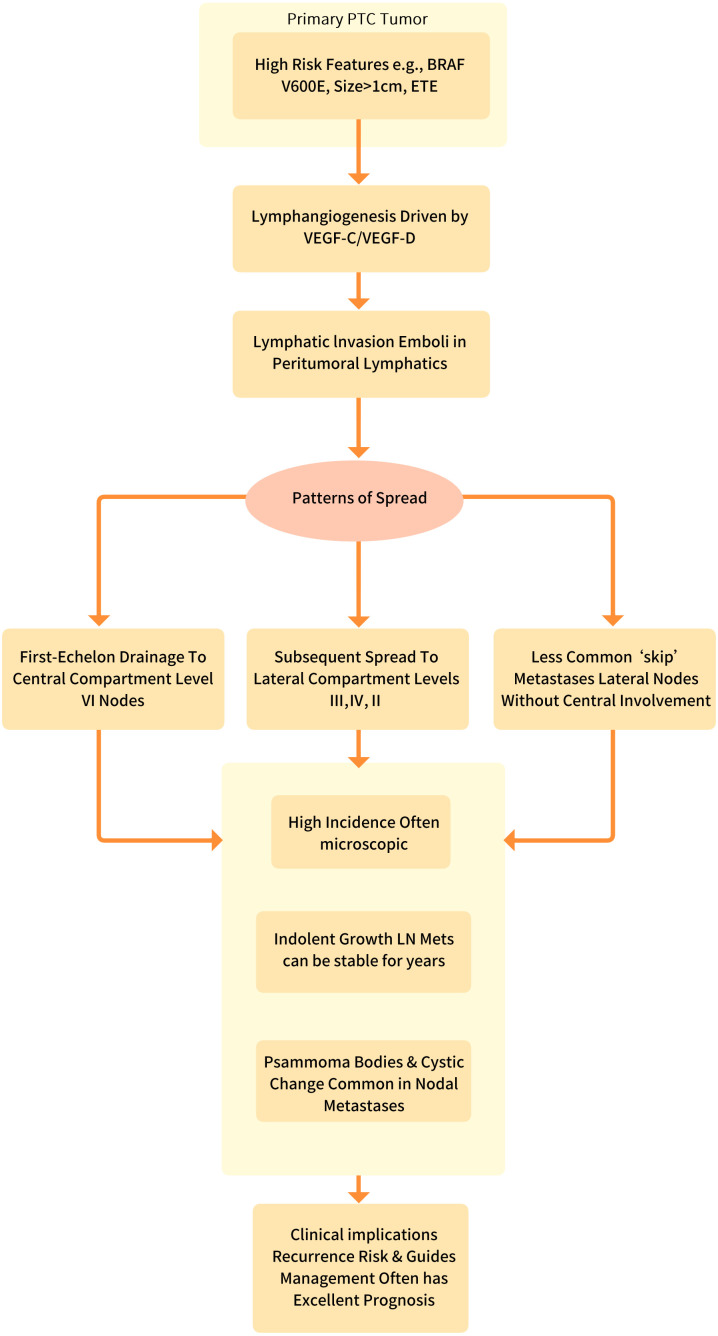
The lymphatic metastasis mechanism of papillary thyroid carcinoma.

Lymphangiogenesis plays a crucial role in the lymphatic metastasis of papillary thyroid carcinoma (PTC), primarily driven by the VEGF-C/VEGFR-3 signaling axis. In this pathway, tumor-derived VEGF-C binds to VEGFR-3 on lymphatic endothelial cells, activating downstream signaling that promotes lymphatic vessel formation and remodeling (17, 18) (**Figure 2**). Key molecular drivers such as the BRAF V600E mutation and RET/PTC rearrangements significantly enhance VEGF-C expression through MAPK pathway activation, establishing an aggressive phenotype with heightened lymphatic metastatic potential (31–33).

The tumor microenvironment further amplifies this process through stromal components including cancer-associated fibroblasts and tumor-associated macrophages. These cells secrete additional lymphangiogenic factors such as PDGF-BB and IL-1β, collectively increasing lymphatic vessel density and creating permissive conditions for tumor dissemination (34–36).

Clinically, enhanced lymphangiogenesis correlates strongly with adverse pathological features including lymph node metastasis, extranodal extension, and aggressive histological variants such as tall cell PTC (37–40). Quantification of lymphatic vessel density through immunohistochemical markers has emerged as a valuable prognostic tool, predicting both recurrence risk and potential resistance to radioactive iodine therapy. These insights are driving the development of novel therapeutic approaches targeting lymphangiogenic pathways, including VEGFR-3 inhibitors and combination immunotherapies. Simultaneously, advances in imaging techniques like indocyanine green lymphography are improving the precision of metastatic node detection during surgery. This comprehensive understanding of lymphangiogenesis mechanisms provides a foundation for refined risk stratification and targeted interventions to disrupt lymphatic spread in high-risk PTC cases.

VEGF-C signaling not only directly suppresses the cytotoxic function of CD8+ T cells and natural killer cells but also fosters the recruitment and differentiation of immunosuppressive regulatory T cells (Tregs) and M2-like macrophages into the metastatic niche (41, 42). This creates an “immunosuppressive shield” around lymphatic vessels, facilitating the immune-evasive survival and transit of tumor cells. Furthermore, the concept of lymphatic endothelial plasticity has come to the fore. Activated lymphatic endothelial cells (LECs), upon VEGFR-3 stimulation, are not passive conduits but active participants in metastasis (43, 44). They can undergo transcriptomic reprogramming, upregulating adhesion molecules to create a “sticky” surface for tumor cell adhesion, and secreting chemokines that act as a homing beacon for CCR7-expressing PTC cells (45). Most intriguingly, recent single-cell RNA sequencing studies suggest that a subpopulation of LECs may even exhibit a hybrid, pro-metastatic phenotype, blurring the lines between traditional endothelial and mesenchymal states (46). Therefore, future therapeutic strategies must evolve from simply blocking vessel growth to disrupting this multifaceted signaling hub—by combining VEGFR-3 inhibition with immune checkpoint blockers to dismantle the immunosuppressive shield, or by targeting the specific adhesion and chemotaxis programs driven by plastic LECs.

In summary, the lymphatic metastasis of PTC is not a passive dissemination but a highly organized, multi-step cascade driven by dynamic tumor-host interactions. This process, encompassing EMT-driven cellular plasticity, VEGF-C/VEGFR-3-mediated lymphangiogenesis and immune reprogramming, chemokine-guided homing, and eventual intravasation, represents the current mechanistic paradigm. However, this linear model fails to fully capture the spatiotemporal heterogeneity and adaptive resilience of metastatic cells.

### 2.2 Metastatic cascade

The metastatic cascade of papillary thyroid carcinoma (PTC) begins with intratumoral lymphangiogenesis, a process driven by tumor-secreted factors that promote the growth of new lymphatic vessels within and around the tumor (47–49). Key mediators include VEGF-C and VEGF-D, which bind to VEGFR-3 on lymphatic endothelial cells (LECs), stimulating their proliferation and migration (50–53). This process is further amplified by BRAF V600E mutations and RET/PTC rearrangements, which upregulate lymphangiogenic signaling pathways. The resulting increased lymphatic vessel density (LVD) creates conduits for tumor cell escape, facilitated by cancer-associated fibroblasts (CAFs) and tumor-associated macrophages (TAMs), which remodel the extracellular matrix (ECM) to enhance lymphatic invasion.

Following lymphangiogenesis, lymphovascular invasion (LVI) occurs, where PTC cells detach from the primary tumor and enter lymphatic channels (54–56). This step is mediated by epithelial-mesenchymal transition (EMT), characterized by the downregulation of E-cadherin and upregulation of N-cadherin and vimentin, enabling tumor cells to adopt a migratory phenotype. Podoplanin (D2-40) and LYVE-1, markers of lymphatic endothelium, are often overexpressed in these invading cells. LVI is a critical prognostic factor, as its presence correlates with higher lymph node metastasis rates and increased risk of recurrence. Additionally, immune evasion mechanisms allow tumor cells to survive within lymphatic vessels and avoid detection by host defenses.

The final stage, lymph node colonization, involves the arrest and growth of tumor cells in regional lymph nodes, particularly in the central compartment (Level VI) before spreading to lateral compartments (Levels II-V) (57, 58). Tumor cells adapt to the nodal microenvironment by interacting with immune cells and stromal components, creating a pre-metastatic niche that supports their survival and proliferation. Extranodal extension (ENE), where tumor cells breach the lymph node capsule, is a marker of aggressive disease and is associated with poorer outcomes (59–61). Clinically, this cascade underscores the importance of early detection through imaging (e.g., ultrasound, CT) and targeted therapies (e.g., VEGFR-3 inhibitors, immunotherapy) to disrupt metastatic progression and improve patient outcomes.

## 3 Anatomical patterns of lymphatic spread

The lymphatic drainage of papillary thyroid carcinoma (PTC) follows predictable primary drainage pathways that correlate with tumor location within the thyroid gland (62–64). Upper pole tumors primarily drain to level II/III (upper and mid-jugular) nodes, while mid-lobe tumors preferentially metastasize to level VI (central compartment) nodes, including the pretracheal, paratracheal, and prelaryngeal (Delphian) nodes. Lower pole tumors and isthmic lesions typically spread first to level VI and level IV (lower jugular) nodes, with isthmic tumors showing particular propensity for bilateral central compartment involvement due to midline lymphatic crossover. These anatomical relationships explain why central neck dissection (level VI) forms the foundation of surgical management in PTC.

Metastatic distribution in PTC demonstrates a characteristic progression pattern, with central compartment (level VI) involvement occurring in 40-65% of cases, representing the first echelon of nodal spread (65–71). From there, metastasis typically extends to lateral compartment nodes (levels II-V) in 25-45% of cases, with levels III and IV being most frequently affected. Approximately 5-15% of patients exhibit skip metastases, where lateral compartment nodes are involved without central compartment disease, a phenomenon particularly associated with upper pole tumors that may drain directly into the jugular chain through alternative lymphatic pathways. The presence of mediastinal (level VII) nodes occurs in 5-15% of cases and generally indicates more advanced disease.

Unusual metastatic patterns occur in specific clinical scenarios and warrant special consideration. Retropharyngeal node metastasis (<2% of cases) typically arises from superior pole tumors that drain via lymphatic channels following the superior thyroid artery (72–74). Parotid node involvement is exceptionally rare and suggests tumor extension along the external jugular lymphatic system (75). Even more uncommon are axillary node metastases, which may represent either aberrant drainage or systemic dissemination. These atypical patterns often correlate with aggressive histological variants (e.g., tall cell, diffuse sclerosing) and frequently coincide with extrathyroidal extension and distant metastases, necessitating comprehensive imaging evaluation in such cases.

In summary, the anatomical patterns of lymphatic diffusion in PTC demonstrate a generally predictable progression from the central to lateral compartments, yet are frequently punctuated by non-canonical pathways such as skip metastases to the lateral neck. These observed routes represent more than passive anatomical drainage; they are the physical manifestation of a complex interplay between pre-existing lymphatic architecture, dynamic tumor-driven lymphangiogenesis, and the molecular characteristics of the tumor cells themselves.

## 4 Clinicopathological correlates

The clinicopathological correlates of lymphatic metastasis in papillary thyroid carcinoma (PTC) reveal distinct patterns where tumor size >2 cm, multifocality, and extrathyroidal extension serve as independent predictors of nodal involvement, with BRAF V600E mutation (present in 40-60% of cases) demonstrating particularly strong association with both central and lateral compartment metastases through VEGF-C-mediated lymphangiogenesis (14, 76–78). Aggressive variants including tall cell (LNM rate 70-90%) and diffuse sclerosing (LNM rate 80-100%) subtypes show exceptional metastatic propensity, often presenting with extranodal extension and higher Tg levels (>50 ng/mL) postoperatively. While younger age (<55 years) paradoxically correlates with increased lymph node metastasis frequency (50-80%), these patients maintain excellent survival, whereas older patients with nodal disease exhibit significantly higher recurrence rates (30-40%) and disease-specific mortality, particularly when accompanied by TERT promoter mutations or >5 metastatic nodes (79–81). The RET/PTC rearrangements (10-20%) are uniquely associated with early, multifocal lymphatic spread often involving skip metastases, while lymphovascular invasion on histopathology emerges as the strongest microscopic predictor of nodal burden, informing decisions regarding prophylactic central neck dissection and radioactive iodine adjuvant therapy in clinically node-negative cases (13). These features collectively underscore the dual role of lymphatic metastases as both a staging marker and therapeutic target in PTC management.

While the phenomenon of “skip metastases” is increasingly recognized in PTC, the literature presents considerable heterogeneity regarding its reported incidence and clinical significance. This discrepancy stems not from the inaccuracy of the concept itself, but from a constellation of methodological and biological variables that are often unaccounted for in individual studies. Firstly, studies employing routine histopathology alone are likely to under-detect micrometastases in central nodes (level VI), thereby artificially inflating the observed rate of “skip” metastases to lateral compartments (levels III-IV). Secondly, surgical and anatomical rigor must be considered. Inconsistent surgical practices, particularly in the completeness of central neck dissection, and unappreciated anatomical variations in lymphatic drainage pathways can confound the true pattern of spread. Most critically, emerging evidence points to molecular phenotypic determinism. Therefore, the debate over the prevalence of skip metastases is a reflection of the field’s evolving technical capabilities and its nascent understanding of biologically distinct PTC subtypes with unique metastatic tropisms. Future research must stratify patients based on these technical and molecular parameters to resolve existing controversies and accurately define the true clinical impact of this metastatic pattern.

## 5 Therapeutic management

The primary treatment for clinically evident lymphatic metastasis (cN1) in PTC involves therapeutic compartment-oriented neck dissection, with central compartment (level VI) dissection being mandatory for involved nodes and selective lateral neck dissection (levels II-V) for confirmed lateral metastases (82–87). For microscopic nodal disease discovered postoperatively (pN1), the extent of intervention depends on risk stratification: radioactive iodine (RAI) therapy (30–150 mCi) is typically recommended for patients with >3 metastatic nodes, extranodal extension, or nodes >2 cm, while observation may be considered for low-volume (≤5 microscopic nodes, all <0.2 cm) central compartment disease (88, 89). Emerging techniques like indocyanine green (ICG) fluorescence-guided surgery are improving intraoperative nodal detection, particularly for recurrent disease, while postoperative stimulated thyroglobulin levels (>10 ng/mL) and neck ultrasound guide decisions regarding adjuvant therapy and surveillance intervals (90–92).

The management of the central neck compartment in clinically node-negative (cN0) papillary thyroid carcinoma remains one of the most debated topics in endocrine surgery, with recent guidelines reflecting evolving evidence. The 2022 ATA Guidelines update maintains a nuanced position, stating that prophylactic central neck dissection (pCND) may be considered in patients with advanced primary tumors (T3-T4) or when the information would guide further treatment decisions, but recommends against routine pCND in low-risk cN0 disease (4). This cautious approach stems from accumulating evidence demonstrating that while pCND improves staging accuracy and potentially reduces locoregional recurrence, these benefits must be weighed against increased surgical morbidity. Emerging data suggest that selective use of pCND based on sophisticated preoperative risk stratification may optimize outcomes. Factors including tumor size >4 cm, multifocality, male gender, and specific sonographic features have been associated with higher rates of occult central neck metastases (>60% in high-risk subgroups) (4). Additionally, the evolving concept of “selective pCND” – targeting the prelaryngeal, pretracheal, and paratracheal compartments based on tumor location – may reduce morbidity while maintaining oncological efficacy. Future directions include developing validated nomograms incorporating clinicopathological and molecular features to identify patients most likely to benefit from pCND, potentially resolving the current clinical equipoise.

Radioactive iodine (RAI) therapy represents a fundamental component in the management of PTC with lymphatic metastasis, though its efficacy demonstrates considerable heterogeneity across patient subgroups. Molecular stratification reveals particularly compromised efficacy in BRAF V600E-mutant tumors, which demonstrate significantly reduced RAI avidity due to impaired sodium-iodide symporter expression (93, 94). Recent advances in redifferentiation strategies show promising results, with MEK inhibitors such as selumetinib restoring RAI uptake in approximately 40-50% of previously RAI-refractory patients and achieving objective response rates of 40-50% when combined with RAI therapy in phase II trials (95, 96).

For RAI-refractory lymphatic metastases, molecular-targeted therapies (lenvatinib, sorafenib) that inhibit VEGFR signaling show 50-65% response rates in reducing nodal burden, while selective RET inhibitors (selpercatinib) demonstrate particular efficacy in RET-altered PTC (97, 98). Novel approaches under investigation include combination strategies pairing PD-1 inhibitors with anti-angiogenics to overcome the immunosuppressive nodal microenvironment, and dose-escalated RAI (200 mCi) with lithium augmentation for poorly differentiated nodal foci. The management paradigm continues evolving toward risk-adapted approaches, where molecular profiling (BRAF/RET/TERT status) and serial thyroglobulin monitoring increasingly guide therapeutic intensity, balancing oncologic control against treatment-related morbidity in this generally indolent metastatic pattern.

In summary, the current therapeutic management of PTC lymphatic metastasis remains predominantly surgical, guided by clinicopathological risk stratification. The cornerstone of treatment involves compartment-oriented lymph node dissection, with the extent—ranging from prophylactic central neck dissection to therapeutic lateral neck dissection—determined by pre-operative imaging and intraoperative findings. Adjuvant radioiodine therapy may be employed for patients with structurally or biochemically incomplete responses. While this paradigm effectively controls disease in most patients, it is inherently reactive and carries non-trivial risks of surgical morbidity, including hypoparathyroidism and nerve injury, underscoring the need for more refined approaches.

## 6 Prognostic implications

The prognostic implications of lymphatic metastasis in papillary thyroid carcinoma (PTC) demonstrate a complex dichotomy where microscopic nodal disease (≤5 involved nodes, all <2 cm without extranodal extension) typically has minimal impact on survival (10-year disease-specific survival >95%), while macroscopic metastases (>3 cm), extranodal extension, or extensive nodal burden (>5 nodes) increase recurrence risk 3–5 fold and correlate with reduced survival in older patients (>55 years) (2, 4, 99, 100). The biological behavior varies significantly by molecular profile, with BRAF V600E-positive nodal metastases showing 2.5 times higher locoregional recurrence rates compared to BRAF wild-type tumors, and TERT promoter mutations conferring particular risk for progression to distant metastases (HR 4.2). While lateral compartment involvement (N1b) traditionally portends worse outcomes than central compartment disease (N1a), contemporary studies suggest the metastatic nodal size and extranodal extension status represent more robust prognostic factors than compartmental location alone. Importantly, the prognostic significance of lymph node metastasis appears modulated by patient age, with younger patients (<55 years) maintaining excellent long-term outcomes despite frequent nodal involvement, whereas older patients with similar nodal burden face significantly higher disease-specific mortality (15-20% at 10 years), highlighting the importance of age-stratified risk assessment in PTC management (4, 6).

## 7 Future directions

Future directions in understanding and managing lymphatic metastasis of papillary thyroid carcinoma (PTC) will focus on precision medicine approaches integrating molecular profiling with advanced imaging to enable early detection and personalized therapy. Emerging liquid biopsy techniques, including circulating tumor DNA (ctDNA) and exosomal miRNA analysis, may allow non-invasive monitoring of minimal nodal disease and prediction of treatment response. Artificial intelligence-assisted ultrasound and lymphotropic nanoparticle-enhanced MRI are being developed to improve detection of micrometastases (<2mm) currently missed by conventional imaging. Therapeutically, next-generation VEGFR-3-specific tyrosine kinase inhibitors and lymphatic-targeted drug delivery systems (nanoparticles carrying BRAF V600E siRNA) show promise in preclinical models to selectively disrupt metastatic spread while sparing normal lymphatics. Immunologically, STING pathway agonists combined with PD-1 inhibitors are being tested to overcome the immunosuppressive nodal microenvironment in BRAF-mutant PTC. Additionally, prophylactic lymphatic mapping using fluorescent tracers may redefine surgical approaches for high-risk patients. These innovations, coupled with organoid models of lymphatic metastasis for drug testing, aim to transform PTC management from reactive to proactive strategies, potentially preventing lymphatic dissemination before clinical manifestation.

Despite comprehensive characterization of individual pathways in PTC lymphatic metastasis, several paradigm-shifting questions remain inadequately addressed, representing critical frontiers for future research. A primary knowledge gap lies in the dynamic spatiotemporal evolution of the pre-metastatic niche: how do PTC-derived exosomes and non-coding RNAs educate distant lymphatic basins even before tumor cell arrival? Furthermore, the field suffers from a mechanistic decoupling between driver mutations (e.g., BRAFV600E) and the resultant metastatic phenotype. Not all BRAF-mutant tumors metastasize, suggesting the existence of epigenetic and microenvironmental “rheostats” that modulate metastatic propensity—a area ripe for single-cell multi-omics investigation. Clinically, the overreliance on static, primary tumor-based biomarkers fails to capture the adaptive plasticity of metastatic cells. We propose that future efforts must pivot towards mapping the metastatic “interactome”—the continuous crosstalk between circulating tumor cells, lymphatic endothelial cells, and immunosuppressive macrophages within the lymphatic fluid itself. Finally, translating these mechanisms into clinic requires developing context-specific therapeutic vulnerabilities that target not just the initiation but the maintenance of lymphatic metastases, potentially through disrupting metabolic symbiosis or mechanical sensing pathways. Addressing these gaps will move the field beyond descriptive correlation towards a mechanistic, predictive, and ultimately targetable understanding of PTC lymphatic spread.

## 8 Conclusions

Lymphatic metastasis in papillary thyroid carcinoma (PTC) exhibits distinct biological and clinical patterns that significantly influence disease management. The conventional stepwise progression from central (Level VI) to lateral (Levels II-V) nodal compartments reflects orderly lymphatic drainage, observed in 60-75% of cases, with tumor size (>2 cm), extrathyroidal extension, and BRAF V600E mutation serving as key drivers. In contrast, skip metastases (5-20% of cases)—bypassing the central compartment—often arise from upper pole tumors or RET/PTC-rearranged variants, challenging standard surgical approaches and necessitating tailored imaging protocols. Notably, limited nodal metastases (≤5 micrometastases) demonstrate indolent behavior with 10-year survival >95%, while bulky (>3 cm) or extranodal-involved metastases correlate with 30-40% recurrence rates, particularly in TERT-mutant or older patients. Emerging strategies prioritize precision detection and molecular-guided therapies to address this spectrum of metastatic behavior. Future efforts should refine risk-adapted paradigms that distinguish inconsequential nodal disease from high-risk metastases warranting aggressive intervention, ultimately optimizing outcomes while minimizing overtreatment.
